# Resting metabolic rate and skeletal muscle SERCA and Na^+^/K^+^ ATPase activities are not affected by fish oil supplementation in healthy older adults

**DOI:** 10.14814/phy2.14408

**Published:** 2020-04-27

**Authors:** Sebastian Jannas‐Vela, Shannon L. Klingel, Daniel T. Cervone, Kate A. Wickham, George J. F. Heigenhauser, David M. Mutch, Graham P. Holloway, Lawrence L. Spriet

**Affiliations:** ^1^ Department of Human Health and Nutritional Sciences University of Guelph Guelph ON Canada; ^2^ Exercise Science Laboratory School of Kinesiology Faculty of Medicine Universidad Finis Terrae Santiago Chile; ^3^ Department of Medicine McMaster University Hamilton ON Canada

**Keywords:** Na^+^/K^+^ ATPase, omega‐3, resting metabolic rate, SERCA, skeletal muscle

## Abstract

Omega‐3 polyunsaturated fatty acids (PUFAs) have unique properties purported to influence several aspects of metabolism, including energy expenditure and protein function. Supplementing with n‐3 PUFAs may increase whole‐body resting metabolic rate (RMR), by enhancing Na^+^/K^+^ ATPase (NKA) activity and reducing the efficiency of sarcoplasmic reticulum (SR) Ca^2+^ ATPase (SERCA) activity by inducing a Ca^2+^ leak‐pump cycle**.** The purpose of this study was to examine the effects of fish oil (FO) on RMR, substrate oxidation, and skeletal muscle SERCA and NKA pump function in healthy older individuals. Subjects (*n* = 16 females; *n* = 8 males; 65 ± 1 years) were randomly assigned into groups supplemented with either olive oil (OO) (5 g/day) or FO (5 g/day) containing 2 g/day eicosapentaenoic acid and 1 g/day docosahexaenoic acid for 12 weeks. Participants visited the laboratory for RMR and substrate oxidation measurements after an overnight fast at weeks 0 and 12. Skeletal muscle biopsies were taken during weeks 0 and 12 for analysis of NKA and SERCA function and protein content. There was a main effect of time with decrease in RMR (5%) and fat oxidation (18%) in both the supplementation groups. The kinetic parameters of SERCA and NKA maximal activity, as well as the expression of SR and NKA proteins, were not affected after OO and FO supplementation. In conclusion, these results suggest that FO supplementation is not effective in altering RMR, substrate oxidation, and skeletal muscle SERCA and NKA protein levels and activities, in healthy older men and women.

## Graphical abstract

This figure resumes the main finding of the study which is that fish oil did not affect SERCA and Na^+^/K^+^ ATPase activities.

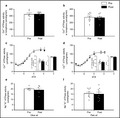

## 1 INTRODUCTION

Resting metabolic rate (RMR) is defined as the amount of energy required to maintain the body's normal metabolic activity at rest in the postabsorptive state (Cunningham, 1980). It accounts for 50%–80% of total energy expenditure each day, and decreases ~1%–2% per decade after 20 years of age (Manini, 2010). In older adults, this reduction has been linked to decreased lean body mass (LBM), primarily skeletal muscle, and to a decreased rate of energy expenditure in organs and tissues (Manini, 2010; Wang et al., 2010). Previous studies have shown that reduced RMR is a potential risk factor for weight gain (Bogardus et al., 1986; Ravussin et al., 1988; Rice et al., 1996). Therefore, developing nutritional interventions to maintain or restore metabolic function in older adults are warranted.

In skeletal muscle, the sarcoplasmic reticulum (SR) Ca^2+^ ATPase (SERCA) and Na^+^/K^+^ ATPase (NKA) are two enzymes that utilize the energy derived from the hydrolysis of ATP to transport ions against a concentration gradient. Specifically, the SERCA pump transports 2 Ca^2+^ ions across the SR membrane, while the NKA pump transports 3 Na^+^ and 2 K^+^ ions across the plasma and transverse tubule membranes (Periasamy & Kalyanasundaram, 2007; Pirkmajer & Chibalin, 2016). Together these enzymes have been shown to contribute up to ~50% of skeletal muscle resting energy expenditure and to be affected by omega‐3 (n‐3) polyunsaturated fatty acids (PUFAs). Previous studies examining the effects of n‐3 PUFAs on skeletal muscle energy metabolism have reported increased rates of protein synthesis in older adults (Lalia et al., 2017; Smith et al., 2011), increased NKA maximal activity in rodent heart tissue membranes with high levels of docosahexaenoic acid (DHA; n‐3 PUFA) (Turner, Else, & Hulbert, 2003), and decreased efficiency and increased permeability of rodent skeletal muscle SERCA with DHA supplementation (Fajardo et al., 2015). Altogether these adaptations have been proposed to lead to increases in whole‐body RMR (Fajardo et al., 2015; Hulbert, 2007).

However, in older adults, there is limited and inconsistent evidence regarding the effects of n‐3 PUFA on whole‐body resting energy metabolism. A study from our group observed a significant increase in resting oxygen consumption (+14%) and fat oxidation (+19%) in community‐dwelling sedentary females (*n* = 12; 60–76 years) after a 12‐week period of fish oil (FO) supplementation (5 g/day), while no changes were observed in the olive oil (OO) control group (Logan & Spriet, 2015). In contrast, a recent study in sedentary older adults (7 females and 5 males; 65–85 years) reported no effect of a 4‐month n‐3 PUFA supplementation (3.9 g/day of eicosapentaenoic acid [EPA] and DHA) on RMR and substrate oxidation (Lalia et al., 2017). Although the evidence regarding increases in resting energy expenditure after n‐3 PUFA supplementation in older adults is limited, it is tempting to speculate that a portion of the increases in resting energy expenditure in the former study may have occurred as a result of changes in protein synthesis, and the activities of NKA and SERCA, in skeletal muscle.

Thus, the purpose of this study was to examine the effects of FO supplementation on RMR, substrate oxidation, and skeletal muscle SERCA and NKA pump function in a group of healthy older females and males. It was hypothesized that RMR, as well as skeletal muscle SERCA and NKA activities, would increase with FO supplementation.

## 2 MATERIAL AND METHODS

### 2.1 Subjects

**Figure 1:**
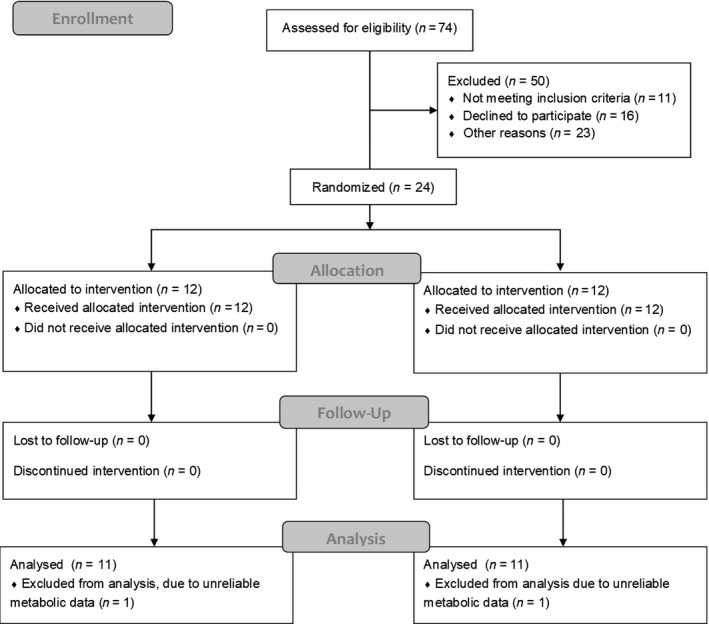
Consort figure illustrating participant flow through the study

Healthy physically active (5 days/week; 60–90 min/day) older adult males (*n* = 8) and females (*n* = 16) volunteered to participate in this study (Figure 1). A written informed consent was received from each subject following a detailed explanation of the experimental protocol and any associated risks. Subjects who met the following inclusion criteria were recruited into the study: (a) between the ages 60 and 75 years; (b) consumed two meals or less of fish per week; (c) did not take an n‐3 PUFA supplement; (d) took no prescription medications or low dose medications for chronic conditions such as, diabetes, hypertension, hypercholesterolemia, and hormone replacement therapy; and (e) absence of any self‐reported medical diagnoses that entailed functional impairment. Participants were instructed to maintain consistent dietary and exercise habits throughout the study. The study was approved by the University of Guelph and McMaster University Research Ethics Boards, and registered in http://clinicaltrials.gov (#NCT02338401).

### 2.2 Study design

Participants visited the laboratory (Department of Human Health and Nutritional Sciences, University of Guelph) in the morning following an overnight fast and performed a familiarization trial where RMR and substrate oxidation were measured. After 1–5 days, a dual energy X‐ray absorptiometry (DXA) scan was performed. Subjects were subsequently matched for RMR, age, and body composition and were randomly assigned in a double‐blinded manner to the FO or OO supplement group. Participants then visited the laboratory in the morning at two different time points (during weeks 0 and 12) at the same time of day (±2 hr) for measurements of RMR and substrate oxidation. Fasted blood samples were taken immediately after these measurements. In addition, resting skeletal muscle biopsies were performed at weeks 0 and 12 in a subset of FO (4 males and 6 females) and OO (3 males and 4 females) participants.

### 2.3 Supplementation

The OO group (*n* = 12, 8 females) supplemented with 5 capsules of OO (1 g OO/capsule [66% oleic acid, 9% palmitic acid, and 4% linoleic acid], Swanson Health Products) for a total of 5 g OO/day. The FO group (*n* = 12, 8 females) was supplemented with 5 capsules/day of Omega‐3 Complete (1 g FO/capsule, Jamieson Laboratories Ltd.), where each capsule provided 0.4 g of EPA and 0.2 g of DHA in triglyceride (TG) form, for a total of 2 g EPA and 1 g DHA/day. Subjects in both the groups were encouraged to take capsules throughout the day (e.g., take 2 at breakfast and 3 at dinner). To promote supplement compliance, subjects were only given 2–3 weeks of capsules at a time. Written and oral reminders were also given periodically to ensure capsules were consumed, and diet and exercise practices remained consistent throughout the study.

### 2.4 Metabolic measurements

Subjects were asked to refrain from any physical activity, alcohol, and caffeine consumption 24 hr prior to each study visit. For the RMR measurements, subjects arrived at the laboratory following an overnight fast (8–14 hr). Participants were instructed to lie supine on a bed for ~30 min in a quiet, darkened room and were asked to breathe normally through a Hans Rudolph mouthpiece with nose clip during the last 18 min. Respiratory measurements were collected using a metabolic cart (AEI MOXUS II Metabolic System) and the last 10 min of data were analyzed for resting oxygen consumption (VO_2_) and carbon dioxide production (VCO_2_). Respiratory exchange ratio was calculated as VCO_2_/VO_2_. Whole body resting fatty acid (FA) oxidation, carbohydrate (CHO) oxidation, and RMR were calculated using the following equations (Péronnet & Massicotte, 1991):

$$\text{FA oxidation}\left(g/\min\right)=\left[1.695\times {\text{VO}}_{2}\left(L/\min\right)\right]-\left[1.701\times {\text{VCO}}_{2}\left(L/\min\right)\right],$$

$$\text{CHO oxidation}\left(g/\min\right)=\left[4.585\times {\text{VCO}}_{2}\left(L/\min\right)\right]-\left[3.226\times {\text{VO}}_{2}\left(L/\min\right)\right],$$

$$\text{RMR}\left(\text{kcal}\right)={\text{VO}}_{2}\left(L/\min\right)\times \text{RER caloric equivalent}\left(\text{kcal}/L\right)\times \text{time}\left(\min\right).$$

One female participant from each group was removed due to unreliable resting metabolic measurements. The variability for RMR and substrate oxidation measurement is 2%–4% and 15%–25%, respectively (Jannas‐Vela, Roke, Boville, Mutch, & Spriet, 2017).

### 2.5 Blood analysis

Fasted serum samples were sent to LifeLabs Medical Laboratory Services immediately after collection and analyzed for metabolic and inflammatory markers including TG, total cholesterol, low‐density lipoprotein‐cholesterol, high‐density lipoprotein‐cholesterol (HDL‐c), the cholesterol/HDL‐c ratio, glucose, and high‐sensitivity C‐reactive protein (hsCRP).

For FA analysis, a second aliquot of blood was centrifuged to separate plasma and red blood cells (RBCs). These fractions were aliquoted and stored at −80°C until further analysis. The FA profile of RBC samples was measured by gas chromatography, as previously described (Roke et al., 2017). FA peaks were identified by comparison to retention times of FA methyl ester standards. Fatty acids were expressed as a percent of total FAs.

### 2.6 Body composition

Body composition (i.e., body fat, LBM) was measured using DXA (Hologic Discovery Wi). Participants were asked to refrain for 1 hr from food and drink, and were asked to lie on the scanning bed for ~6 min. X‐rays of two different energies were emitted from below the subject and were detected by a moving “arm” above, from head to toe. Prior to use, the DXA was calibrated using a standard calibration block of thermoplastic acrylic resin, according to the manufacturer's instructions.

### 2.7 Muscle biopsies

Two to four resting muscle biopsies (total ~50 to 150 mg) were obtained under local anesthesia (2% lidocaine without epinephrine) from the vastus lateralis muscle, using the percutaneous needle biopsy technique described by Bergstrom (1975). A portion of muscle (~50 mg) was immediately diluted 11:1 (vol/wt) in ice‐cold homogenizing buffer containing (in mM) 0.2 PMSF, 250 sucrose, 5 HEPES, and 0.2% NaN3 (pH 7.5), using a hand‐held glass homogenizer (Jannas‐Vela et al., 2019). The homogenate was separated into 2–5 aliquots, flash‐frozen in liquid nitrogen, and stored at −80°C for future analyses of ATPase activities and western blotting of selected proteins. The bicinchoninic acid assay was performed to determine the protein content of homogenates.

#### 2.7.1 Measurement of SERCA activity

Total SERCA activity was measured using a fluorometric assay (Jannas‐Vela et al., 2019). The reaction buffer contained (in mM) 200 KCl, 20 HEPES, 15 MgCl_2_, 1 EGTA, 10 NaN_3_, 5 ATP, and 10 phospho(enol)pyruvate (pH 7.0 at 37°C). Before starting the reaction, 18 U/ml lactate dehydrogenase (LDH), 18 U/ml pyruvate kinase (PK), 5 μl homogenate, and 0.2 mM NADH were added to a cuvette containing ~1.5 ml of reaction buffer in the presence or absence of 40 μM ionophore (A23187, Sigma). Assays were performed in duplicate at 37°C using a fluorometer (Lumina; Thermo Scientific, Fisher) set with an excitation wavelength of 340 nm and an emission wavelength of 460 nm. SERCA activity was assessed by adding 15 μl of 10 mM CaCl_2_. SERCA activity increases with free Ca^2+^ concentrations ([Ca^2+^]f) until a plateau occurs, indicating maximal activity. The [Ca^2+^]f corresponding to each CaCl_2_ addition was determined using an online calculator (*Ca‐Mg‐ATP‐EGTA Calculator* *v* *1.0*, 2017) with the following constants for input fields: ionic strength 0.28, pH 7.0, temperature 37°C, 1 mM EGTA, 5 mM ATP and 15 mM Mg^2+^. The rate of ATPase activity was calculated from the negative slope (fluorescence second^−1^) of NADH from a standard curve established with the same reaction conditions. SERCA activity was obtained by subtracting ATPase activity in the presence of 40 μM cyclopiazonic acid (which completely inhibits SERCA) from total ATPase activity at different [Ca^2+^]f.

The kinetic properties of SERCA that were assessed included the maximal activity (*V*max), the [Ca^2+^]f needed to obtain half‐maximal activity, and the Hill coefficient. To obtain the kinetic properties of the enzyme, SERCA activity was plotted against the negative logarithm of [Ca^2+^]f (pCa). The *V*max represented the peak value, whereas the pCa50 represented the pCa obtained from a sigmoid fit of the data, which yields 50% of *V*max. The Hill coefficient was determined through a nonlinear regression (variable slope) with Prism software (GraphPad Software, Inc.) by using a portion of the curve that corresponded to between 20% and 80% of maximal activity, using the following sigmoidal dose–response equation:

$$Y=Y_{\text{bot}}+\left(Y_{\text{top}}-Y_{\text{bot}}\right)/\left(1+10^{\left(\log{\text{Ca}}_{50}-X\right)}\times n_{H}\right),$$

where *Y* is the plateau, *Y*_bot_ is the value at the bottom of the plateau and *Y*_top_ is the value at the top, log Ca_50_ is the concentration that gives a response halfway between *Y*_bot_ and *Y*_top_, and *n*_H_ is the Hill coefficient.

To estimate SERCA permeability, an ionophore ratio was calculated in which maximal SERCA activity with ionophore was divided by the maximal SERCA activity without ionophore (Jannas‐Vela et al., 2019). One participant from the OO group was excluded due to an inability to accurately measure SERCA activity.

### 2.8 Measurement of NKA activity

Measurement of NKA activity was performed with an enzyme‐coupled method using an ATP regenerating assay (Jannas‐Vela et al., 2019). After freeze‐thawing muscle homogenates 5 times, an aliquot (10 μl) was incubated at 37°C in a buffer containing (in mM) 40 Tris HCl, 15 KCl, 1 EGTA, 5 MgCl_2_, 1.5 phospho(enol)pyruvate, 3 ATP (pH 7.4), in the presence of 18 U/ml of PK and LDH, 25 μM of blebbistatin, and 0.3 mM NADH. Assays were performed in duplicate using a fluorometer (Lumina; Thermo Scientific, Fisher) set with an excitation wavelength of 340 nm and an emission wavelength of 460 nm. Maximal NKA activity was measured by addition of 80 mM NaCl. The rate of NKA activity was calculated from the negative slope (fluorescence second^−1^) of NADH from a standard curve established with the same reaction conditions. NKA maximal activity was obtained by subtracting ATPase activity in the presence and absence of 2 mM ouabain (ouab), using the following equation:

$$\text{NKA activity}\left(\mu \text{mol}/\min/\text{g protein}\right)=\left(\left(Y_{\left[\text{Na}\right]}-Y_{\left[\text{ouab}\right]}\right)/\text{m}/\mu \text{g of protein}\right)\times 1000,$$

where *Y*_[Na]_ is the change in fluorescence in the presence of 80 mM Na, *Y*_[ouab]_ is the change in fluorescence after addition of 2 mM ouab, and ‘m’ is the slope obtained from the standard curve. One participant from each supplement group was excluded due to an inability to accurately measure NKA activity.

### 2.9 Western blotting

Western blot analyses were performed on muscle homogenates using methods described previously (Gerling, Whitfield, Mukai, & Spriet, 2014; Herbst et al., 2014). Proteins (10 μg) were separated by electrophoresis on a 10% or 15% SDS‐polyacrylamide gel, and then transferred to a polyvinylidene difluoride membrane. Membranes were blocked in 5% nonfat milk in Tris‐buffered saline containing 0.1% Tween‐20 (TBS‐T) for 1 hr at room temperature, and then incubated overnight at 4°C in primary antibody diluted in 5% bovine serum albumin. After three washes in TBS‐T, membranes were incubated at room temperature for 1 hr with the corresponding secondary antibody, detected using enhanced chemiluminescence (Perkin Elmer) and quantified by densitometry (Alpha Innotech Fluorchem HD2, Fisher Scientific).

The following commercially available antibodies were used: SERCA1a (cat. no. CaF2‐5D2; Developmental Studies Hybridoma Bank [DSHB], University of Iowa), SERCA2a (cat. no. ab2861; Abcam), calsequestrin 1 (CSQ1) (cat. no. ab191564; Abcam), CSQ2 (cat. no. ab108289; Abcam), NKA α1 (cat. no. A6F; DSHB), NKA α2 (cat. no. 07‐674; MilliporeSigma), NKA β1 (cat. no. ab193669; Abcam), NKA β2 (cat. no. ab185210; Abcam), NKA β3 (cat. no. 610992; BD Biosciences), and α‐tubulin (cat. no. 2148; Cell Signaling). All samples for a given target were transferred onto a single membrane to limit variation. All samples were normalized to α‐tubulin.

### 2.10 Statistical Analysis

All data are presented as means ± standard error of the means (*SEM*s). A two‐way repeated measures ANOVA was used to examine the effects of treatment group and time on body composition, blood measurements, RMR, and substrate oxidation. A paired *t* test was used to examine the independent effects of treatment on skeletal muscle ATPase activities and protein expression. Statistical significance was considered as *p* ≤ .05. GraphPad Prism, Version 7.0 (GraphPad Software, Inc.) was used for all statistical analyses and all data were checked for normality before analysis.

## 3 RESULTS

### 3.1 Subject characteristics, blood analysis, and RMR

Participants were well matched for pre‐intervention physical characteristics between the OO and FO supplementation groups (Table 1). After 12 weeks of FO and OO supplementation, there was no change in diet, body mass, LBM and body fat (Table 1).

**Table 1:** Subject characteristics

	Olive oil (*n* = 11)		Fish oil (*n* = 11)	
	Week 0	Week 12	Week 0	Week 12
Age (y)	66 ± 1	—	65 ± 1	—
Height (m)	1.68 ± 0.03	—	1.66 ± 0.03	—
Body mass (kg)	69.7 ± 3.8	70.4 ± 4.1	71.5 ± 3.7	71.3 ± 3.7
Body fat (%)	33.1 ± 2.5	33.7 ± 2.6	34.7 ± 1.9	35.2 ± 1.9
Lean body mass (%)	67.0 ± 2.5	66.3 ± 2.6	65.3 ± 1.9	64.8 ± 1.9
VO_2_ (ml/min)^a^	226 ± 37	215 ± 37	230 ± 31	215 ± 30
VCO_2_ (ml/min)	175 ± 29	174 ± 33	182 ± 31	174 ± 29
RER^a^	0.775 ± 0.056	0.810 ± 0.059	0.789 ± 0.047	0.808 ± 0.054
Diet (kcal)	1,864 ± 147	1,731 ± 110	1,784 ± 91	1,783 ± 402
Carbohydrate (%)	51 ± 3	49 ± 2	50 ± 3	48 ± 3
Fat (%)	32 ± 2	33 ± 3	32 ± 3	32 ± 3
Protein (%)	17 ± 2	18 ± 2	18 ± 1	20 ± 2

Note

All values are means ± *SEM*.

Abbreviation: RER, respiratory exchange ratio.

^a^ Main effect of time (*p* < .05).

There were no differences in baseline blood measurements between the OO and FO supplementation groups. After supplementation, plasma levels of hsCRP were decreased (*p* < .05) and HDL‐c was increased (*p* < .05) in the FO group (Table 2). Additionally, RBC levels of EPA and DHA were increased (*p* < .0001) after FO supplementation from 0.72 ± 0.05% to 3.43 ± 0.36%, and from 6.39 ± 0.24% to 8.28 ± 0.16%, respectively (Table 2). RBC levels of EPA and DHA were not affected after OO supplementation.

**Table 2:** Blood measures

	Olive oil (*n* = 11)		Fish oil (*n* = 10)	
	Week 0	Week 12	Week 0	Week 12
Glucose (mmol/L)	4.91 ± 0.08	4.85 ± 0.10	5.72 ± 0.56	5.93 ± 0.46
Triglycerides (mmol/L)^a^	1.33 ± 0.22	1.24 ± 0.12	0.94 ± 0.08	0.80 ± 0.12
Cholesterol (mmol/L)	5.90 ± 0.45	5.50 ± 0.44	5.27 ± 0.39	5.34 ± 0.52
HDL‐c (mmol/L)	1.80 ± 0.13	1.70 ± 0.12	1.80 ± 0.11	1.99 ± 0.16^b^
LDL‐c (mmol/L)	3.50 ± 0.38	3.24 ± 0.37	3.04 ± 0.32	2.98 ± 0.42
hsCRP (mg/L)	1.82 ± 0.78	1.98 ± 0.76	1.79 ± 0.48	0.81 ± 0.19^b^
EPA (% of total FAs)	0.72 ± 0.04	0.70 ± 0.04	0.72 ± 0.05	3.43 ± 0.36^b^
DHA (% of total FAs)	5.98 ± 0.31	5.96 ± 0.31	6.39 ± 0.24	8.28 ± 0.16^b^

Note

All values are means ± *SEM*.

Abbreviations: DHA, docosahexaenoic acid; EPA, eicosapentaenoic acid; FAs, fatty acids; HDL‐c, high‐density lipoprotein–cholesterol; hsCRP, high sensitivity C‐reactive protein; LDL‐c, low‐density lipoprotein‐cholesterol.

^a^ Main effect of group (*p* < .05).

^b^ Significant difference within group (*p* < .05).

**Figure 2:**
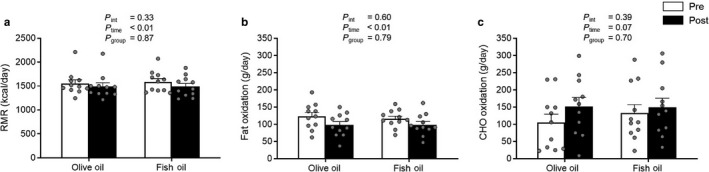
Resting metabolic rate (RMR) (a), carbohydrate (CHO) oxidation (b), and fat oxidation (c) before (Pre‐white bars) and after (Post‐black bars) olive oil and fish oil supplementation. Values are reported as means ± *SEM*. Data were analyzed by 2‐factor repeated‐measures ANOVA

Baseline values of RMR and substrate oxidation were not different between the OO and FO supplementation groups. After supplementation, there were no significant interaction effects in RMR and substrate oxidation (*p* > .30). However, there was a main effect of time with decrease in RMR (*p* < .01) and fat oxidation (*p* < .01) in both the supplementation groups (Figure 2a,b). CHO oxidation was not affected after OO or FO supplementation (Figure 2c).

### 3.2 Activity and expression of SERCA and NKA proteins

**Figure 3:**
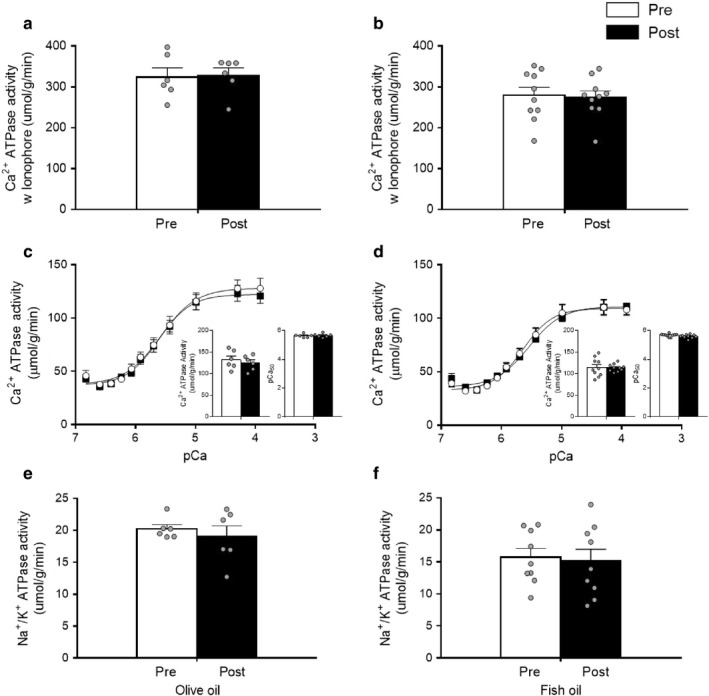
Maximal sarcoplasmic reticulum Ca^2+^ ATPase (SERCA) pump activity with ionophore before (Pre‐white bars) and after (Post‐black bars) supplementation with olive oil (*n* = 6) (a) and fish oil (*n* = 10) (b). Kinetic curve showing SERCA activity, maximal SERCA pump activity (inset), pCa50 (inset) Pre and Post supplementation with olive oil (*n* = 6) (c) and fish oil (*n* = 10) (d). Na^+^/K^+^ ATPase maximal activity Pre and Post supplementation with olive oil (*n* = 6) (e) and fish oil (*n* = 9) (f). Values are reported as means ± *SEM*. Data were analyzed by paired *t* test

**Figure 4:**
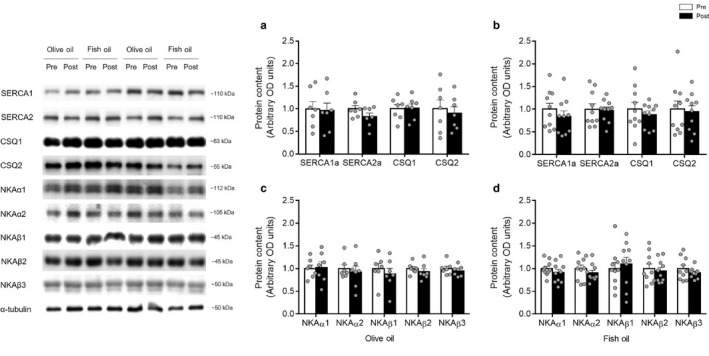
Expression of protein targets contributing to sarcoplasmic reticulum Ca^2+^ATPase (SERCA) and Na^+^/K^+^ ATPase (NKA) activities before (Pre‐white bars) and after (Post‐black bars) supplementation with olive oil (*n* = 7) and fish oil (*n* = 10). No effect of supplementation on the expression of SERCA 1a and 2a, calsequestrin 1 and 2 (CSQ1, CSQ2) (a, b); NKA_α1_, NKA_α2_, NKA_β1_, NKA_β2_, NKA_β3_ (c, d). Values are reported as means ± *SEM*. Data were analyzed by paired *t* test

To obtain a ratio of SR permeability, measurements of SERCA pump maximal activity in skeletal muscle homogenates were performed with and without the presence of the Ca^2+^ ionophore A23187. SERCA pump maximal activity with the ionophore was not affected with either OO (Pre: 324 ± 22; Post: 328 ± 18) or FO (Pre: 279 ± 19; Post: 274 ± 16) supplementation (Figure 3a,b). Similarly, SERCA activity without the presence of the ionophore was not affected with either OO (Pre: 132 ± 9; Post: 125 ± 7) or FO (Pre: 114 ± 7; Post: 114 ± 3) supplementation (Figure 3c,d). Consequently, the calculated ionophore ratio was similar after OO (Pre: 2.46 ± 0.03; Post: 2.63 ± 0.08) and FO (Pre: 2.44 ± 0.09; Post: 2.41 ± 0.13) supplementation. The enzyme kinetics of SERCA were also unaffected by supplementation, as pCa_50_ and Hill coefficients remained similar in both the groups (Figure 3c,d). Likewise, NKA maximal activity was not affected with OO (Pre: 20 ± 1; Post: Pre: 19 ± 2) or FO (Pre: 16 ± 2; Post: Pre: 15 ± 2) supplementation (Figure 3e,f). Since SERCA and NKA activities were not affected by OO and FO, we checked whether the protein levels of SR calcium handling proteins and NKA isoforms remained unaltered by OO and FO. As expected, the levels of SERCA, CSQ, and NKA isoforms did not change after OO and FO supplementation (Figure 4).

## 4 DISCUSSION

In the present study, we provide evidence that supplementation with FO (2 g/day EPA; 1 g/day DHA) has no effect on whole‐body RMR and substrate oxidation in healthy older adults. Further, we also demonstrated that FO supplementation did not affect skeletal muscle SERCA permeability, SERCA and NKA activities, or the content of SR calcium handling and NKA proteins. Altogether, these results demonstrate that RMR and skeletal muscle NKA and SERCA activities are not affected by n‐3 PUFA supplementation in older adults.

After the 12‐week supplementation period, we observed decrease in RMR and fat oxidation, and no change in CHO oxidation, with both OO and FO. These results suggest that n‐3 PUFA supplementation had no effect on RMR and substrate oxidation, consistent with recent investigations which reported no change on RMR and substrate oxidation after n‐3 PUFA supplementation in healthy young individuals (Jannas‐Vela et al., 2017; Noreen et al., 2010), sedentary older adults (Lalia et al., 2017), overweight individuals (Kratz, Callahan, Yang, Matthys, & Weigle, 2009), and insulin resistant patients (Lalia et al., 2015). In contrast, a study by our research group in untrained older females (*n* = 12; 60–76 years) showed a significant increase in RMR (14%) and fat oxidation (19%) following 12 weeks of FO supplementation (2 g/day EPA, 1 g/day DHA), while the OO control group reported no change on either RMR or substrate oxidation (Logan & Spriet, 2015). A potential explanation for these discrepancies could be attributed to subject characteristics, as the present study used physically active older males and females compared to the past study that used only untrained older females. When excluding the male participants (*n* = 4) in the present study, BMI and body fat percentages of the female subjects were ~15% lower than those reported in females from the previous study by Logan & Spriet (2015). As RMR and substrate oxidation remained unchanged after supplementation in the present study, it may suggest that healthy older females are more resistant to changes in energy expenditure and body composition by FO. Future studies are warranted in older adults to determine whether resting energy metabolism is affected by health status or sex in response to FO supplementation.

In‐line with previous work (Eslick, Howe, Smith, Priest, & Bensoussan, 2009; Pischon et al., 2003; Pluess et al., 2007), we found that the plasma levels of HDL‐c increased and that the plasma levels of hsCRP decreased after FO supplementation. However, to our surprise, we observed that FO supplementation had no effect on the plasma levels of TG. It is likely that this occurred because the baseline values of TG were considerably lower (~0.90 mg/dl) than the normal range (≤1.50 mg/dl). This unexpected finding has also been reported after long‐term FO n‐3 PUFA supplementation in clinical patients with normal TG levels (Mita et al., 2007; Tokudome et al., 2015).

While evidence is mounting to suggest that n‐3 PUFAs do not regulate whole‐body energy metabolism in humans, it is possible that the molecular changes affecting RMR after FO supplementation cannot be detected by the current methods of indirect calorimetry (Jannas‐Vela et al., 2017). Rodent work has shown that n‐3 PUFAs have the capacity to affect ATPase enzymes (Fajardo et al., 2015); however, to date, there are no studies in humans that have examined if n‐3 PUFAs regulate the expression and activities of SERCA and NKA pumps. We observed no effect of FO supplementation on the kinetic parameters of SERCA and the expression of SR calcium handling proteins in healthy older adults. The current finding contradicts a recent study reporting increased permeability and decreased efficiency of the SERCA pump after 8 weeks of DHA supplementation in rodent skeletal muscle (Fajardo et al., 2015). It is possible that the discrepancies between studies could be due to the model and type of supplement used, as the study by Fajardo et al. (2015) fed young rodents (5 months) for 8 weeks a diet high in DHA, whereas the present study supplemented older men and women with a mixture of EPA and DHA. In agreement, other studies in rodents that used FO showed either no effect on SERCA activity (Stubbs & Kisielewski, 1990) or increased efficiency of Ca^2+^ uptake/release across the SR membrane (Dulloo, Decrouy, & Chinet, 1994).

We also observed that NKA maximal activity and protein levels were not affected after FO supplementation. This result contradicts previous findings in rodents showing that heart microsome membrane DHA content was positively associated with maximal NKA activity (Hulbert, 2007; Turner et al., 2003). It is possible that these differences were due to the use of rodent versus human samples, the tissues examined (heart microsomes vs. skeletal muscle), and the methodologies employed to determine NKA maximal activity. It is also possible that n‐3 PUFAs were not incorporated into skeletal muscle membranes, as we only detected increases of EPA and DHA in RBC membranes. However, this is unlikely as it was recently demonstrated that EPA and DHA were incorporated into RBC and whole muscle phospholipids after 4 weeks of FO supplementation (McGlory et al., 2014). Furthermore, previous research in older men and women of similar age reported increased content of EPA and DHA in muscle membranes after only 8 weeks of supplementation (Smith et al., 2011). Therefore, we strongly believe that the changes in RBC membrane composition reflect the changes that occurred in skeletal muscle.

We recently showed distinct incorporation of n‐3 PUFAs into human skeletal muscle plasma and mitochondrial membranes after FO supplementation, where the sarcolemma was less responsive than whole muscle and mitochondria (Gerling et al., 2019). SERCA pumps reside in the SR, while NKA pumps reside in the sarcolemma, transverse tubules, and intracellular vesicles (Clausen, 2003). It is possible that these fractions were less responsive to FO supplementation, and the changes in membrane composition were not sufficient to alter SERCA and NKA function. Future studies are required to determine the effects of FO on individual membrane fractions and their effect on SERCA and NKA function.

The rationale to suggest that n‐3 PUFAs could increase energy expenditure were based on past studies in rodents that reported a positive association between DHA content in cell membranes and increased RMR (Hulbert, 2007), increased membrane leak pump cycles (Fajardo et al., 2015; Hulbert, 2007), and increased activity of membrane bound proteins (Turner et al., 2003). In contrast, the present study observed no effects on whole body RMR or skeletal muscle SERCA permeability, and SERCA and NKA activities in healthy older adults. Furthermore, recent research has reported that mitochondrial leak (state 4 respiration) assessed with permeabilized muscle fibers was not changed after supplementation period with n‐3 PUFAs in healthy young males (Herbst et al., 2014) and insulin resistant individuals (Lalia et al., 2015). Altogether, these results further corroborate that the potential mechanisms that lead to increased RMR with n‐3 PUFA supplementation in rodents were unaltered in humans.

In conclusion, this study has demonstrated that supplementation with FO in healthy, active older adults had no effect on RMR and whole‐body substrate oxidation. In addition, supplementation with FO did not affect SERCA and NKA activities or the expression of SR calcium handling and NKA proteins in skeletal muscle. Altogether these results show that FO does not affect RMR, and skeletal muscle SERCA and NKA activities and protein levels in healthy older adults.

## CONFLICT OF INTEREST

The authors declare no conflict of interest.

## AUTHOR CONTRIBUTIONS

It should say:SJ‐V, GJFH, GPH, DMM, and LLS contributed to the conception of the study. SJ‐V, SLK, DTC, KAW, GJFH, GPH, and LLS assisted with measurements. SJ‐V, GPH, and LLS analyzed and interpreted the data, and wrote the first draft of the manuscript. All authors read and approved the final manuscript.
